# Dynamic changes in DNA methylation during embryonic and postnatal development of an altricial wild bird

**DOI:** 10.1002/ece3.5480

**Published:** 2019-08-17

**Authors:** Hannah Watson, Pablo Salmón, Caroline Isaksson

**Affiliations:** ^1^ Evolutionary Ecology, Biology Department Lund University Lund Sweden; ^2^ Institute of Biodiversity, Animal Health and Comparative Medicine University of Glasgow Glasgow UK

**Keywords:** birds, developmental plasticity, developmental programming, DNA methylation, epigenetics, phenotypic development

## Abstract

DNA methylation could shape phenotypic responses to environmental cues and underlie developmental plasticity. Environmentally induced changes in DNA methylation during development can give rise to stable phenotypic traits and thus affect fitness. In the laboratory, it has been shown that the vertebrate methylome undergoes dynamic reprogramming during development, creating a critical window for environmentally induced epigenetic modifications. Studies of DNA methylation in the wild are lacking, yet are essential for understanding how genes and the environment interact to affect phenotypic development and ultimately fitness. Furthermore, our knowledge of the establishment of methylation patterns during development in birds is limited. We quantified genome‐wide DNA methylation at various stages of embryonic and postnatal development in an altricial passerine bird, the great tit *Parus major*. While, there was no change in global DNA methylation in embryonic tissue during the second half of embryonic development, a twofold increase in DNA methylation in blood occurred between 6 and 15 days posthatch. Though not directly comparable, DNA methylation levels were higher in the blood of nestlings compared with embryonic tissue at any stage of prenatal development. This provides the first evidence that DNA methylation undergoes global change during development in a wild bird, supporting the hypothesis that methylation mediates phenotypic development. Furthermore, the plasticity of DNA methylation demonstrated during late postnatal development, in the present study, suggests a wide window during which DNA methylation could be sensitive to environmental influences. This is particularly important for our understanding of the mechanisms by which early‐life conditions influence later‐life performance. While, we found no evidence for differences in genome‐wide methylation in relation to habitat of origin, environmental variation is likely to be an important driver of variation in methylation at specific loci.

## 1 INTRODUCTION

There is increasing interest among ecologists and evolutionary biologists in the potential for epigenetic mechanisms to shape environmentally induced phenotypic responses (Angers, Castonguay, & Massicotte, 2010; Bossdorf, Richards, & Pigliucci, 2008; Hu & Barrett, 2017; Verhoeven, VonHoldt, & Sork, 2016). Accumulating evidence demonstrates that epigenetic marks can be induced or removed in response to environmental cues in natural populations of plants and animals (Gugger, Fitz‐Gibbon, Pellegrini, & Sork, 2016; Herrera & Bazaga, 2011; Lea, Altmann, Alberts, & Tung, 2016). Furthermore, it has been suggested that the epigenome is most sensitive to environmental influences during development (Jirtle & Skinner, 2007), giving rise to stable behavioral or physiological traits (Waterland & Jirtle, 2003; Weaver et al., 2004), which can subsequently affect fitness (Rubenstein et al., 2016). Epigenetic modifications could thus promote phenotypic plasticity and facilitate rapid adaptation (Jablonka & Raz, 2009), which may be especially important in changing or novel environments (Hu & Barrett, 2017).

It is well understood that the early‐life environment plays a fundamental role in shaping the phenotype, which can lead to permanent changes in physiology, behavior and morphology (Monaghan, 2008). Epigenetics are a prime candidate for mediating this developmental plasticity and linking early‐life conditions with later‐life effects (Gluckman, Hanson, Buklijas, Low, & Beedle, 2009; Gluckman, Hanson, Spencer, & Bateson, 2005). DNA methylation—the most widely studied epigenetic mark—plays a crucial role in regulating gene expression and genomic stability (Klose & Bird, 2006; Weber et al., 2007). It has been shown in a number of vertebrates, in the laboratory, that the methylome undergoes reprogramming during development. Genome‐wide demethylation is followed by de novo methylation in the early stages of embryogenesis (Kafri et al., 1992; Li, Guo, Zhang, Gao, & Guo, 2014; Mhanni & McGowan, 2004; Monk, Boubelik, & Lehnert, 1987; Stancheva, El‐Maarri, Walter, Niveleau, & Meehan, 2002; Usui et al., 2009). However, the extent and schedule of reprogramming varies widely, and the plasticity of the methylome also extends into later stages of development (Gluckman et al., 2009; Simmons, Stringfellow, Glover, Wagle, & Clinton, 2013). The established patterns of methylation are subsequently maintained through mitotic cell division and can thus create permanent changes in gene expression and consequently stable phenotypic traits (Jablonka & Raz, 2009; Weaver et al., 2004). This cycle of epigenetic reprogramming appears to be critical in directing embryonic development and determining patterns of DNA methylation in somatic cells (Bird, 2002). Indeed, dysregulation of methylation during development can lead to imprinting disorders (Waterland, Lin, Smith, & Jirtle, 2006) or developmental arrest (Li, Bestor, & Jaenisch, 1992).

Studies of variation in DNA methylation in free‐living vertebrates in an ecological context are lacking (Bentz, Sirman, Wada, Navara, & Hood, 2016; Laine et al., 2016; but see Lea et al., 2016; Liebl, Schrey, Richards, & Martin, 2013; Riyahi, Sanchez‐Delgado, Calafell, Monk, & Senar, 2015; Viitaniemi et al., 2019), especially during the critical period of development (but see Rubenstein et al., 2016; Sheldon, Schrey, Ragsdale, & Griffith, 2018). Studies of epigenetics in the wild are essential for understanding the fitness consequences of environmentally induced epigenetic modifications, and they could offer valuable insight into the resilience of populations to environmental change. Dynamic genome‐wide changes in DNA methylation have been demonstrated during embryonic and postnatal development in the precocial domestic chicken, revealing temporal and tissue‐specific changes in methylation patterns (Li et al., 2014; Usui et al., 2009). However, it is important to note that these two studies have examined selected tissues and developmental stages, and the picture, in the chicken, is far from complete.

Since altricial birds are at a much earlier stage of development when they hatch, compared with precocial birds, it is reasonable to expect that the establishment of the methylome could be different to that of precocial birds. To the best of our knowledge, no study has attempted to describe establishment of the methylome in an altricial bird, though methylation has been shown to be sensitive to environmental factors during development (Rubenstein et al., 2016; Sheldon et al., 2018). In the present study, we quantify changes in global DNA methylation during development in the great tit *Parus major*. DNA methylation was quantified in embryonic tissue at embryonic days 1, 3, 6, and 12, and in the blood of nestlings at 6 and 15 days posthatch. The great tit is a small passerine bird, with altricial development, and is a model species in evolutionary ecology. Using embryos and nestlings originating from both urban and rural environments, we also investigated the potential for environmentally induced variation in genome‐wide DNA methylation levels.

## 2 MATERIALS AND METHODS

### 2.1 Study system and field data collection

Great tits produce a clutch of typically 6–9 eggs, which hatch following an incubation period of *c*. 13–14 days incubation. The altricial nestlings hatch naked, with eyes closed, and they require warmth and food to be provided by the parents; nestlings remain in the nest until fledging at 17–19 days. Eggs (*n* = 89) were collected from great tit nests (*n* = 56) during 21 April–6 May 2014. Eggs originated from an urban population (*n* = 44) in the city of Malmö (population: 300,000) and a rural population (*n* = 45) in the forest of Vombs fure, located 32 km from Malmö and with <5 inhabitants/km^2^ in surrounding areas. The third and fourth eggs in the laying sequence were collected on the day of laying and stored for up to 5 days (median ± *SD* = 2.0 ± 0.95) at 12°C and in darkness, prior to artificial incubation (see below). Nests were followed through to fledging of young. Repeated blood samples were collected from 27 nestlings (nests = 26; 12 urban, 14 rural) at 6 days (*n* = 26) and 15 days (*n* = 23) posthatch from a random subset of nests from which eggs were collected.

### 2.2 Egg incubation procedure

Eggs were randomly assigned to one of four incubation treatments on embryonic day 0; eggs were incubated in multiples of 24 hr and sacrificed at either embryonic day 1, 3, 6, or 12 (from here on denoted as E1, E3, E6, and E12, respectively). While methylation has a genetic component, large variation is likely to be introduced due to differential maternal allocation of hormones and nutrients among eggs in the clutch. We therefore opted to allocate two eggs per clutch to the same treatment group, as opposed to allocating four eggs per clutch across all incubation groups, and simultaneously minimize impacts at the individual‐ and population‐level. Eggs across all treatment groups were representative of the range of egg‐collection dates and storage times. Incubators (Ruvmax) were kept indoors in a dark, climate‐controlled room. A divider rotates around a central axis, pushing eggs around the egg plate, ensuring that eggs were moved every few minutes. Upon removal from incubators, eggs were transferred to a freezer at −50°C. Egg mass was recorded before and after incubation. Most eggs were incubated in incubator A; eleven eggs were incubated in a second identical incubator B. Conditions within incubators were maintained at 37.04 ± 0.98 and 36.84 ± 1.32°C and 68.3 ± 3.16% and 69.2 ± 3.16% relative humidity in incubators A and B, respectively. Eight eggs failed to develop and were discarded, leaving 81 eggs (53 nests) for analysis.

### 2.3 Quantification of genome‐wide DNA methylation

Eggs were dissected to isolate embryos, which were subsequently washed with PBS and homogenized in 200 μl PBS using a TissueLyser (Qiagen). DNA was isolated from homogenized embryonic tissue and 4 μl whole blood using NucleoSpin Tissue and Blood kits (Macherey‐Nagel), respectively, and according to the manufacturer's protocol. DNA quality was assessed using ultraviolet spectrophotometry (NanoDrop, Thermo Scientific). DNA methylation was quantified by enzyme‐linked immunosorbent assay (EpiGentek MethylFlash P‐1030) according to the manufacturer's protocol with input of 25–100 ng DNA. The range of methylation levels detected among samples was wider than the range of standards available, thus demanding variable DNA input to ensure detection (embryonic tissue: 50–100 ng; nestling blood: 25 ng). The assay volume was always constant, and methylation levels were corrected for mass of DNA. Samples from the same individual were run on the same plate, but due to the fact that many D15 nestling samples fell above the detectable range, a number had to be re‐run at a lower concentration on a separate plate. Samples from different stages were randomly distributed among plates. DNA samples from E1 and E3 were of lower quality (260/280: mean ± *SE* = 1.08 ± 0.03) compared with all other developmental stages (mean ± *SE* = 1.99 ± 0.01); this could be due to lipid contamination as it proved difficult to remove all traces of yolk. Furthermore, all embryos from E1 and E3 fell below detectable levels and could therefore only be assigned the mean detection level of 0.65%, calculated as the limit of the blank (LoB; 1.645 *SD* from the blank). For these reasons, E1 and E3 were excluded from statistical analyses. Average intra‐assay and inter‐assay variation (mean ± *SE*) were 8.3 ± 0.3% and 30 ± 7.8%, respectively.

### 2.4 Statistical analyses

All analyses were performed using lmerTest (Kuznetsova, Brockhoff, & Christensen, 2017) in R 3.2.4 (R Core Team, 2013). Linear mixed models (LMMs) were fitted to logit‐transformed proportions of methylated DNA; models were fitted separately to data from embryonic tissue and nestlings. Starting LMMs included the fixed effects of developmental stage (two‐level factor: 1 [referring to E6 and D6, in embryos and nestlings, respectively] or 2 [referring to E12 and D15]), habitat (two‐level factor: urban or rural), and the interaction between developmental stage and habitat. To control for additional potential sources of variation in DNA methylation of embryonic tissue, primarily as a result of variation in maternal investment, the full LMM also included the fixed effects of laying sequence (two‐level factor: 3rd or 4th), initial egg mass (covariate), and egg‐storage time (five‐level factor: 1–5 days). The LMM fitted to embryonic data included the random effects of nest identity and assay plate (1–6; to control for inter‐plate variability); the LMM fitted to nestling data included the random effects of individual identity nested within nest identity and assay plate (as above). Fixed‐effect terms were eliminated one‐by‐one if *p* > .05 when comparing a reduced model to the original model in likelihood ratio tests; the effects of stage and habitat were retained regardless of significance in a hypothesis‐led approach. The significance of parameter estimates was estimated using conditional *F*‐tests based on Satterthwaite approximation for the denominator degrees of freedom.

## 3 RESULTS

**Figure 1:**
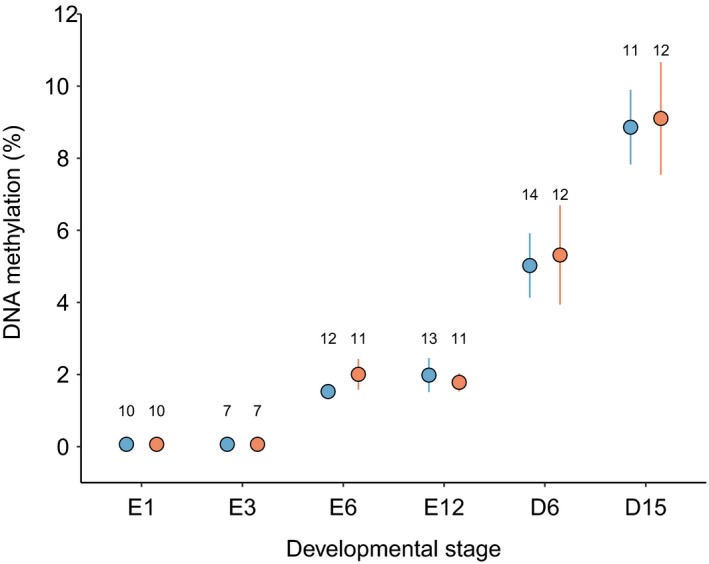
DNA methylation levels (mean ± *SE*) at different stages of embryonic (E) and postnatal (D) development in the altricial great tit *Parus major*. DNA methylation was quantified from whole embryos at embryonic days 1, 3, 6, and 12, and blood of nestlings at 6 and 15 days posthatch. Embryos and nestlings originated from urban (orange) and rural (blue) populations. DNA methylation at E1 and E3 fell below detectable levels and was assigned at the detection limit of the assay (0.65%) and excluded from statistical analyses. Sample sizes are shown for each group

Whole‐genome DNA methylation did not change between embryonic days 6 and 12 (Figure 1; *β*_E12_ = 0.056 ± 0.15, *F*_1,20.9_ = 0.15, *p* = .7), yet genome‐wide DNA methylation in blood of nestlings increased almost twofold between 6 and 15 days posthatch (Figure 1; *β*_D15_ = 0.45 ± 0.14, *F*_1,24.8.0_ = 10.1, *p* = .004). Although analyzed separately, Figure 1 clearly shows that genome‐wide DNA methylation in the blood of posthatch chicks was higher than that of whole‐embryo tissue at any stage of embryonic development. There was no significant effect of habitat on genome‐wide DNA methylation levels at any point during development either in embryonic tissue (*β*_urban_ = −0.011 ± 0.15, *F*_1,20.6_ = 0.0049, *p* = .9) or in nestling blood (*β*_urban_ = −0.12 ± 0.15, *F*_1,23.0_ = 0.60, *p* = .5). Any temporal change between developmental stages was identical in both urban and rural habitats, as indicated by the interaction between habitat and developmental stage (all *p* > .7). Variances (mean ± *SD*) associated with random effects in final LMMs were as follows: embryonic tissue: 0.078 ± 0.28 and 0.081 ± 0.29 for nest and plate, respectively; and, nestling blood: 0.021 ± 0.15, 0.60 ± 0.77 for individual: nest and plate, respectively. There was no significant variation in global DNA methylation as a result of either initial egg mass (*p* = .6) or storage time (*p* = .8). There was a tendency for higher DNA methylation in embryos from fourth laid eggs, compared with those laid third in the egg‐laying sequence (*β*_4th_ = 0.20, *F*_1,20.6_ = 4.3, *p* = .05).

## 4 DISCUSSION

We present the first evidence for within‐individual changes in genome‐wide DNA methylation during development of an altricial bird. Although the evidence for changes during embryonic development is limited, a twofold change in genome‐wide DNA methylation in blood between 6 and 15 days posthatch provides unambiguous proof of modification of tissue‐specific methylation during postnatal development. Since DNA in avian blood primarily comes from erythrocytes, and other cell types contribute little to the total DNA, changes in composition of cell type in the blood are unlikely to explain the observed increase in methylation. The observed developmental change in DNA methylation is of great significance, as it is among the first evidence that DNA methylation could play a fundamental role in shaping phenotypic plasticity during early life in altricial birds (Rubenstein et al., 2016; Sheldon et al., 2018). The ability for methylation patterns to change during development is a key assumption of the hypothesis that DNA methylation shapes the phenotype and mechanistically links early‐life conditions with long‐term effects and mediates developmental programming (Gluckman et al., 2009, 2005). Our results open up new exciting research avenues for investigating the role of the observed changes in global methylation during early life and the uniformity of changes across tissue types. Further studies should seek to determine the stability of the established genome‐wide methylation patterns across an individual's lifespan, which is also a precondition of methylation as a regulator of developmental programming.

The results demonstrate that, similar to other vertebrates (Li et al., 2014; Monk et al., 1987; Stancheva et al., 2002), altricial birds undergo some level of change in DNA methylation during development. In mammals, genome‐wide demethylation is followed by de novo methylation during the early stages of embryogenesis (Kafri et al., 1992; Monk et al., 1987). This is followed by tissue‐ and cell‐specific methylation and demethylation in later stages of development, as shown in mammals (Chen & Riggs, 2011; Simmons et al., 2013; Song et al., 2009) and the chicken (Li et al., 2014). Changes in methylation in postnatal development are corroborated in altricial birds by the changes observed in blood of growing nestlings in the present study. The occurrence of genome‐wide changes in methylation in blood throughout postnatal development is of foremost ecological relevance, as it suggests a wide window during which environmentally induced epigenetic modifications could occur. Furthermore, since blood has been shown to reflect changes occurring in other tissues (Asghar et al., 2016; Watson, Videvall, Andersson, & Isaksson, 2017), the observed changes might not only suggest a wide window of sensitivity to environmentally induced epigenetic changes for this particular tissue, but at the whole‐organism level. Further research—both correlational and experimental—should seek to understand how genome‐wide DNA methylation changes across multiple tissues.

Our data suggest that low levels of genome‐wide DNA methylation during early embryonic development are followed by an increase in global methylation levels during the second half of embryonic development in the great tit. This could indicate that genome‐wide changes in DNA methylation are occurring later in altricial birds, compared with mammals where the major global changes take place in early embryogenesis. Although there is an apparent absence of change in global methylation between days 6 and 12 of embryonic development, tissue‐specific changes could occur, but they cannot be detected when using a homogenized sample of all body tissues. We must also be cautious in drawing any conclusions concerning changes in methylation between early and late embryonic development, due to low DNA quality and undetectable levels of methylation at embryonic days 1 and 3. It is uncertain how the assay is affected by DNA quality, though we believe that the assay would still bind sufficient DNA to yield a detectable signal, if methylation levels were within the detectable range. It would therefore seem likely that levels of methylation in early embryonic development are lower, compared with later stages, but exactly how low and how they change between early embryonic stages is inconclusive.

Given the evidence from other studies for tissue‐specific differences in DNA methylation patterns, we must be cautious in jumping to the conclusion that genome‐wide methylation increases between the end of embryonic development and the posthatch chick, since samples originate from different tissues. However, the twofold increase in DNA methylation in blood between 6 and 15 days posthatch confirms that changes in methylation continue to occur throughout postnatal development. Indeed, in mammals, it has been shown that the plasticity of the epigenome extends into postnatal development and until at least weaning (Song et al., 2009; Waterland et al., 2006). The observed increase in methylation during postnatal development could indicate active methylation of the genome, as genes that were transcriptionally active during early development are switched off. Our results suggest that the window for potential environmentally induced changes in genome‐wide methylation patterns is similarly wide (relative to development, rather than time per se) in altricial birds, compared with mammals. This finding is pertinent to developing our understanding for the potential of epigenetic mechanisms to mediate effects of early‐life conditions.

Finally, neither absolute levels nor developmental changes in genome‐wide DNA methylation differed according to the habitat from which embryos or nestlings originated. Although the study design lacks replicates of habitat types, we have previously reported environmentally induced variation in physiology and fitness‐related traits during development between the two sites (Salmón, Nilsson, Watson, Bensch, & Isaksson, 2017; Salmón, Watson, Nord, & Isaksson, 2018). Since eggs were all incubated in a common environment, any observed habitat differences among embryonic stages would have been driven by genetic or maternal effects. At a whole‐genome level, differences in methylation among nestlings would only be detectable if habitat differences induced constitutive hypo‐ or hyper‐methylation. While such constitutive genome‐wide changes in methylation have previously been demonstrated in association with environmental cues (Sheldon et al., 2018), it is more likely that environmental variation would induce both hypo‐ and hyper‐methylation at select loci. It thus remains possible that gene‐specific environmentally induced changes in methylation patterns could occur between urban‐ and rural‐reared nestlings, despite the fact that we found no evidence for differences in genome‐wide DNA methylation. Developmental conditions are likely to be important in modulating epigenetic variation in the wild (Rubenstein et al., 2016), and much could be learned from studies of how variation in environmental factors, such as pollution, food quality and availability, and ambient temperature, affect DNA methylation patterns.

## CONFLICT OF INTEREST

The authors declare no conflict of interest.

## AUTHOR CONTRIBUTIONS

CI conceived the study; HW & CI designed the study; HW & PS carried out the study; HW performed laboratory and statistical analyses; HW drafted the manuscript with input from CI & PS.

## ETHICAL APPROVAL

All procedures were carried out in accordance with national and European legislation, and approval was granted by the Malmö‐Lund Animal Research Ethics Committee (M454 12:1). Eggs were collected under licence from Naturvårdsverket (NV‐01657‐14).

## Data Availability

Data are deposited in Dryad https://doi.org/10.5061/dryad.2jq5027.
